# Rap GTPase Interactor: A Potential Marker for Cancer Prognosis Following Kidney Transplantation

**DOI:** 10.3389/fonc.2019.00737

**Published:** 2019-08-07

**Authors:** Qiang Fu, Fan Yang, Minxue Liao, Noel J. Feeney, Kevin Deng, Nikolaos Serifis, Liang Wei, Hongji Yang, Kai Chen, Shaoping Deng, James F. Markmann

**Affiliations:** ^1^Organ Transplantation Center, Sichuan Provincial People's Hospital and School of Medicine, University of Electronic Science and Technology of China, Chengdu, China; ^2^Division of Transplantation, Department of Surgery, Massachusetts General Hospital, Harvard Medical School, Boston, MA, United States; ^3^Organ Transplantation Translational Medicine Key Laboratory of Sichuan Province, Chengdu, China; ^4^Women and Children Health Care Center of Luoyang, Luoyang, China

**Keywords:** RADIL, APTX, CD8+ T cells, kidney transplantation, kidney cancer

## Abstract

Post-transplant (post-Tx) kidney cancer has become the second-highest cause of death in kidney recipients. Late diagnosis and treatment are the main reasons for high mortality. Further research into early diagnosis and potential treatment is therefore required. In this current study, through genome-wide RNA-Seq profile analysis of post-Tx malignant blood samples and post-Tx non-malignant control blood samples (CTRL-Tx), we found Rap GTPase Interactor (RADIL) and Aprataxin (APTX) to be the most meaningful markers for cancer diagnosis. Receiver operating characteristic (ROC) curve analysis showed that the area under the curve (AUC) of the RADIL-APTX signature model was 0.92 (*P* < 0.0001). Similarly, the AUC of RADIL alone was 0.91 (*P* < 0.0001) and that of APTX was 0.81 (*P* = 0.001). Additionally, using a semi-supervised method, we found that RADIL alone could better predict malignancies in kidney transplantation recipients than APTX alone. Kaplan-Meier analysis indicated that RADIL was expressed significantly higher in the early stages (I and II) of kidney, liver, stomach, and pancreatic cancer, suggesting the potential use of RADIL in early diagnosis. Multivariable Cox regression analysis found that RADIL together with other factors (including age, stage III, stage IV and CD8+ T cells) play a key role in kidney cancer development. Among those factors, RADIL could promote kidney cancer development (HR > 1, *P* < 0.05) while CD8+ T cells could inhibit kidney cancer development (HR < 1, *P* < 0.05). RADIL may be a new immunotherapy target for kidney cancer post kidney transplantation.

## Introduction

As the optimal treatment for end-stage renal disease (ESRD), renal transplantation can improve the quality of life and overall survival (OS) significantly compared to traditional treatments in ESRD patients (1, 2). However, the increase in cancer frequency in post-transplant (post-Tx) patients may be closely related to the usage of immunosuppressant drugs (3). Diagnoses of post-Tx malignancies usually occur at an advanced stage and lead to poor recipient prognosis (4, 5). Though the pathological grade and tumor node metastasis (TNM) stage are widely applied to cancer diagnosis, accurate grading and sub-staging for some tumors remains controversial. Therefore, identifying new markers for early detection and treatment remain essential to improving the survival of recipients and kidney graft.

Further evidence has revealed the immune system performs important functions in prohibiting the development and progression of cancer (6, 7). Though previous studies suggest that CTLA4, PD-L1/PD-1, and IL-27 can be used as the checkpoints for cancer prognosis and therapy, only a subset of subjects exhibited consistent responses for the target therapy (5, 8). Additionally, various immune signaling cascades are altered due to post-Tx cancer development. Many other factors such as age, race, and drug sensitivity also affect the efficacy of current therapies. These shortcomings in cancer prognosis and therapy necessitate further study of the broader mechanisms of cancer immunity.

Rap GTPase Interactor (RADIL) has emerged as an important Rap effector involved in cell adhesion, migration, and polarity. Rap1a–RADIL signaling performs a critical role in the progression of breast cancer (9). Meanwhile, Aprataxin (APTX) acts as a DNA-repair gene and can repair DNA strand breaks (10). A previous study into the expression of APTX demonstrated that increased APTX expression could regulate the cellular sensitivity to anticancer drug resistance in patients with advanced colorectal cancer (11).

In the current study, we analyzed the genome-wide RNA-Seq profiles of the patients with post-Tx malignancies and tried to identify potential genetic markers for diagnosis and prognosis. We found that RADIL had a high degree of accuracy, and the area under the curve (AUC) of RADIL was 0.91, almost identical to that of the 2-gene signature (APTX – RADIL) model AUC value of 0.92. RADIL may be applicable in immunotherapy targets for the future.

## Methods and Materials

### Microarray Data

Gene expression profiles of GSE94424 and GSE51675, based on GPL6480 Agilent-014850 (Whole Human Genome Microarray 4x44K G4112F) submitted by Giovanni Stallone group are available in the GEO database (5, 12). The two datasets contained 42 peripheral blood mononuclear cell (PBMC) samples, including 10 chronic antibody-mediated rejection after transplantation (CAMR-Tx) samples, 8 control transplant (CTRL-Tx) samples, 8 post-Tx malignancy samples, 8 samples from immunocompetent patients with malignancies (non-Tx malignancies) and 8 healthy samples. The clinical data of those samples were listed in Table S1.

### Identification of Dysregulated Genes in Post-Tx Malignancies

Genes with no expression in more than half of all samples were excluded. Gene expression between the CTRL-Tx group and the post-Tx malignancy group were identified with *t*-test. The genes with a *P* < 0.05 (FDR adjusted) and fold change >2 were considered as differentially expressed analysis (DEGs). The principal component analysis (PCA) was used to evaluate the DEGs with R version 3.4.3.

### Identification of Key DEGs With the Prognostic Score

To identify key DEGs related to prognosis, a semi-supervised method (SPC) was applied with the cut-off of HR > 1 or HR < 1 with *P* < 0.05 (13). The Z-scores of RADIL and APTX were used as the multipliers. The prognostic score was calculated for all 42 patients (16 malign ones and 26 non-malign ones) as follows: Prognostic score = (7.042 × RADIL) + (3.575 × APTX), where 7.042 and 3.575 are the respective Z-scores of RADIL and APTX. Afterwards, we defined CTRL-Tx group and post-Tx malignancy group as the training set, while the CAMR-Tx group, healthy group, and non-Tx malignancy group served as the test set to confirm the effect of RADIL and APTX.

### Expression Analysis of RADIL

To further verify the accuracy of RADIL for diagnosis, Kaplan-Meier with log-rank methods was applied to choose the best cut-offs for separating the samples, which are available in the TCGA data portal, into the high-expression and low-expression groups and to evaluate the OS of the cancer patients first in all stages (I–IV) and then specifically in the early stages (I and II). Patients whose follow-up time was < 30 days from their first appointment were excluded to remove the unrelated causes of death. After the exclusion, 853 samples of kidney cancer containing 223 deceased samples were included, 342 samples of liver cancer with 123 deceased, 342 samples of stomach cancer with 143 deceased, and 170 samples of pancreatic cancer with 90 deceased were included. We also investigated the RADIL expression among different stages using the GEPIA database (14). All the immunohistochemistry of RADIL expression in renal, liver, stomach, and pancreatic cancers were collected from the human protein atlas (www.proteinatlas.org) and the demographic and clinic characteristics were listed in Table S2. The average optical density (AOD) of RADIL was measured (mean ± SD and *P* < 0.05 as significant). In order to evaluate the function of CD8+T cells, which play a key role in inhibiting tumorigenesis, and exclude other exogenous effects on the tumor development, Cox analysis on different types of tumor-infiltrating immune cells and other factors such as age, stage, and race, were performed using multivariable Cox proportional hazard model. The Cox analysis used the TIMER database through which the whole transcriptome RNA-seq data of the tumor tissues from TCGA database were analyzed. The Human Primary Cell Atlas (HPCA) was used as the reference dataset of gene expression profiles of sorted immune cell types (15, 16). The samples with different types of tumor-infiltrating immune cells were included. Finally, 415 kidney cancer samples with 124 deceased were included, 305 liver cancer samples with 101 deceased, 278 stomach cancer samples with 104 deceased, and 165 pancreatic cancer samples with 89 deceased were included (Tables S3–S6). RADIL expression was then validated in GSE2774, in which an immuno-resistant cancer cell line, based on in a mouse model of human papillomavirus (HPV)-associated cervical cancer, was established [Affymetrix Mouse Genome 430 2.0 Array] (17).

### Statistical Analysis

Data were analyzed using R version 3.4.3. or GraphPad Prism version 5. Graft survival was analyzed with Kaplan–Meier and log-rank methods. Differences between experimental groups were analyzed using the Student's *t*-test or ANOVA. *P* < 0.05 was considered statistically significant.

## Results

### Differentially Expressed Genes Identification

**Figure 1:**
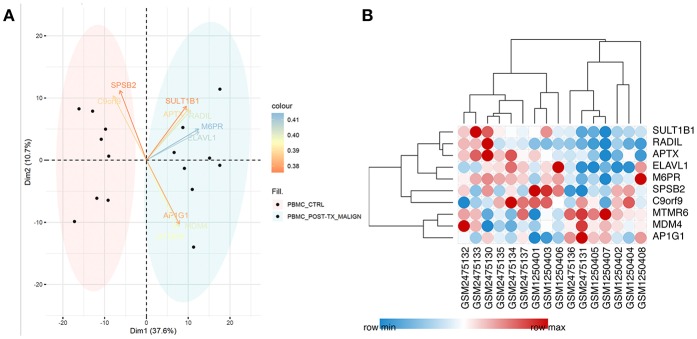
Identification of differentially expressed genes. **(A)** Using PCA methods, the top 10 DEGs were revealed according to the contributed scores between the CTRL-Tx group and post-Tx malignancy group. **(B)** The expression of the DEGs and Pearson correlation analysis.

After filtering out unqualified cases without names, 811 genes between the CTRL-Tx group and post-Tx malignancy group were identified (log |FC| > 1 and *P* < 0.05). Using PCA methods, the top 10 DEGs were revealed according to the contributed scores (Figure 1A). The expression of those genes and Pearson correlation analysis were shown in Figure 1B.

### Establishment of the Gene Prognostic Model

**Figure 2:**
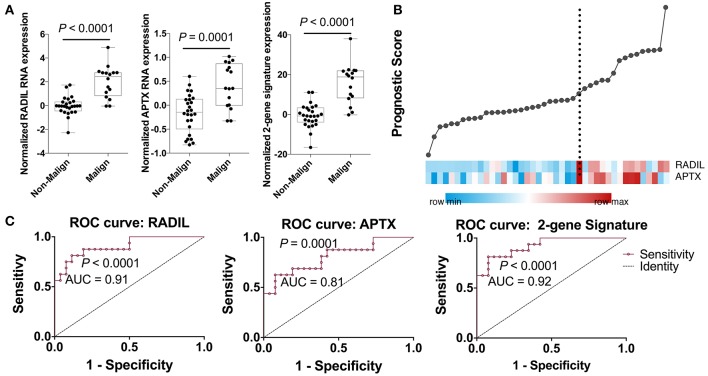
Establishment of the gene prognostic model. **(A)** RADIL and APTX expression between the malign group and non-malign group. **(B)** RADIL-APTX based prognostic model for diagnosis for all those subjects and those with higher prognostic scores demonstrated a tendency toward the expression of high-risk genes. **(C)** ROC curves of RADIL and APTX and 2-gene signature, in which we could find RADIL has a similar AUC (AUC = 0.91) value with that of 2-gene signature (AUC = 0.92).

**Figure 3:**
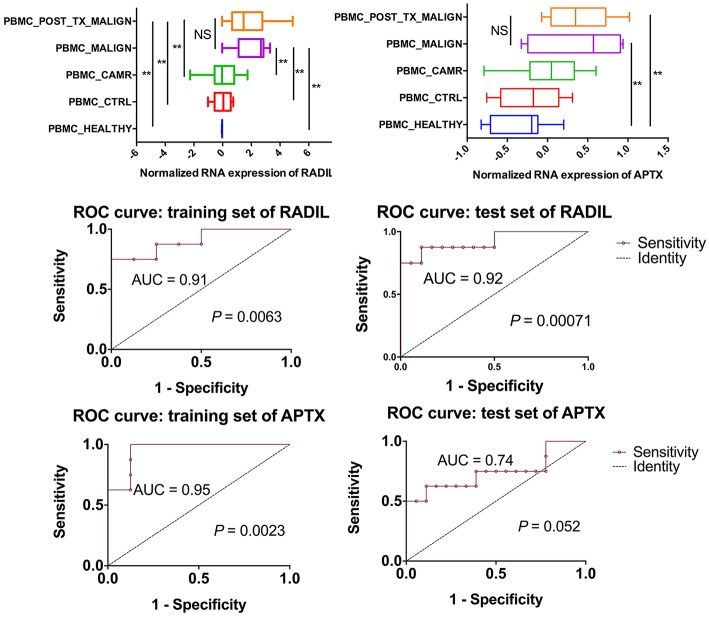
Further analysis on RADIL and APTX. The AUC of APTX was 0.93 in the training group and 0.75 in the test group, suggesting that APTX was not optimal. The AUCs of RADIL in both groups were more than 0.8.

Among those top 10 genes, RADIL and APTX were identified as the DEGs between the malign group and non-malign group (RADIL with *P* < 0.0001 and APTX with *P* = 0.0001) as shown in Figure 2A. Thereafter, we developed a two-gene prognostic model for diagnosis for all 42 subjects, which were ranked according to the prognostic scores. The model demonstrated a positive correlation between prognostic score, and high-risk gene expression (Figure 2B). According to receiver operating characteristic (ROC) curves of RADIL and APTX, we found RADIL has a similar AUC (AUC = 0.91) value with that of 2-gene signature (AUC = 0.92). Out of consideration of the efficient cost and convenience for the test, we analyzed the effect of RADIL and APTX again using SPC to figure out if RADIL could predict cancer alone. As shown in Figure 3, APTX was an inadequate predictor in the test group. Therefore, we thought that RADIL may be appropriate to replace the 2-gene signature for prognosis though the latter had a higher AUC value.

### Validation of the Prognosis of RADIL

**Figure 4:**
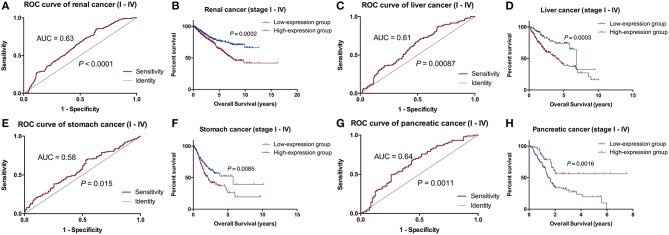
Kaplan-Meier analysis of the common tumors. In the ROC curve analysis, AUC of the patients with renal cancer was 0.63 **(A)**, in liver cancer was 0.61 **(C)**, in stomach cancer was 0.58 **(E)**, and in pancreatic cancer was 0.64 **(G)**. Kaplan-Meier curves indicated that the patients with renal cancer **(B)**, liver cancer **(D)**, and stomach cancer **(F)** in the RADIL high-expression group had poor OS, whereas patients in the low-expression group had positive outcomes; **(H)** pancreatic cancer patients with a high-expression of RADIL had a better OS.

RADIL expression was different among the different tumor stages (*P* < 0.05, Figure S1). Using the optimum cut-off value obtained in ROC curve analysis (Figures 4A,C,E,G), the patients were divided into high- and low- expression groups. This division indicated that renal cancer, liver cancer, and stomach cancer patients in the RADIL high-expression group had poor outcomes, while patients in the RADIL low-expression group had a better OS (*P* < 0.001, Figures 4B,D,F). In contrast, the patients with pancreatic cancer in the RADIL high-expression group possessed a better OS (*P* < 0.001, Figure 4H), suggesting that RADIL may play different roles in different kinds of tumors.

### RADIL Expression in the Early Stage

**Figure 5:**
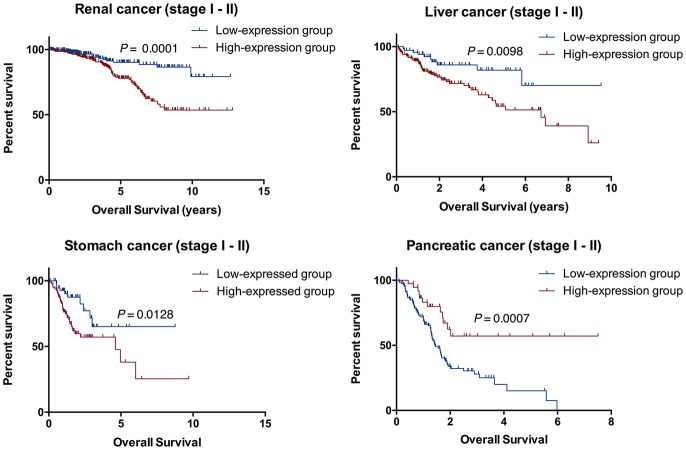
RADIL could be helpful for predicting the survival of patients in stage I and II. AUC calculated with Kaplan-Meier and log-rank methods in the early stage (I and II) patients gave similar results to that of the entire cohort in renal cancer (*P* = 0.0001), liver cancer (*P* = 0.0098), stomach cancer (*P* = 0.0128) and pancreatic cancer (*P* = 0.0007).

To further verify the accuracy of the prognostic model above, Kaplan-Meier and log-rank methods were applied and AUC values were calculated for early-stage patients (I and II). It showed a consistent result to that of the entire cohort in renal cancer (*P* = 0.0001), liver cancer (*P* = 0.0098), stomach cancer (*P* = 0.0128) and pancreatic cancer (*P* = 0.0007, Figure 5). Therefore, RADIL could be conducive to the survival prediction of patients in the early stages.

### Validation of the Expression of RADIL

**Figure 6:**
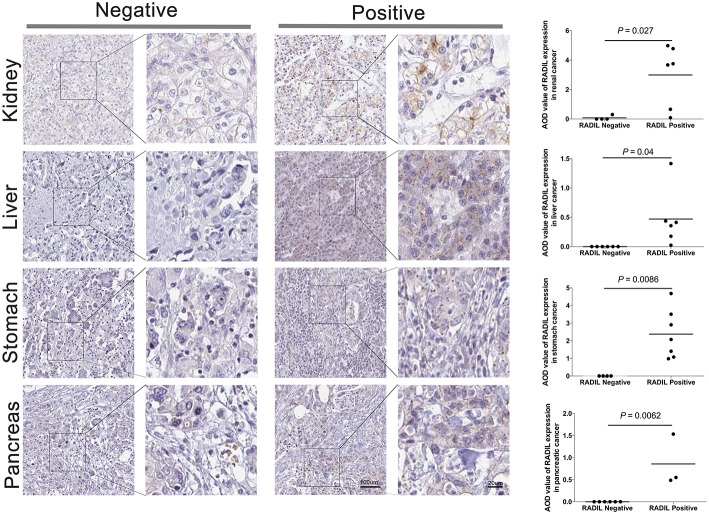
Validation of the prognosis of RADIL. Tumor tissues had higher rates to express RADIL in renal cancer (*P* = 0.027), liver cancer (*P* = 0.04) and stomach cancer (*P* = 0.0086) than that in the negative control groups. While in pancreatic cancer, tumor tissues had a lower RADIL expression (*P* = 0.0062).

Thereafter, RADIL protein expression was identified with immunohistochemistry. AOD results showed a similar trend to that of OS results (Figure 6). Tumor tissues had higher expression of RADIL in renal cancer (*P* = 0.027), liver cancer (*P* = 0.04) and stomach cancer (*P* = 0.0086) compared to the negative control groups. Meanwhile, in pancreatic cancer, tumor tissues had a lower RADIL expression (*P* = 0.0062, Figure 6). When combined with hematoxylin-eosin staining, it was easier to make accurate cancer diagnosis and prognosis (Figure S2).

### RADIL Expression Has a Negative Relationship With Tumor-Infiltrating CD8+ T Cells

**Figure 7:**
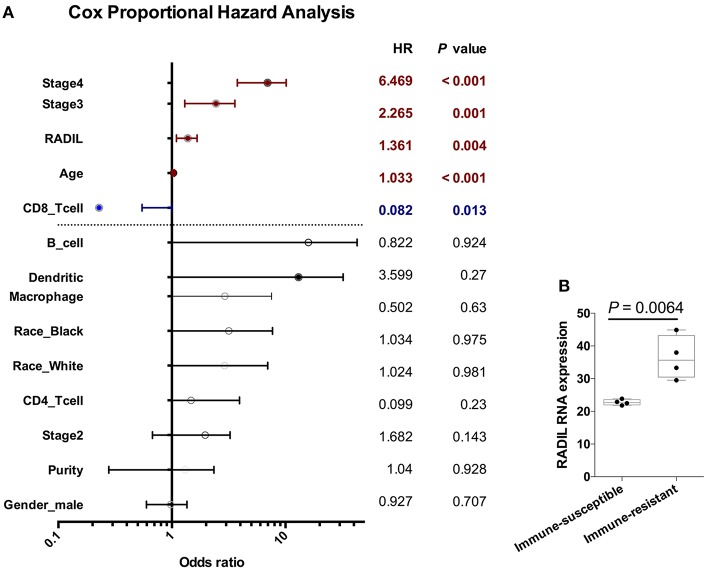
RADIL expression has a negative relationship with CD8+ T cells. **(A)** Cox regression analysis found that RADIL could affect kidney cancer OS along with age (*P* < 0.001), stage III (*P* = 0.001), stage IV (*P* < 0.001) and CD8+ T cells (*P* < 0.001). **(B)** RADIL gene expression increased significantly in murine immune-tolerance tumor cell line compared with the murine immune-susceptible tumor cell line.

Cox regression analysis found that RADIL still affected kidney cancer development along with age (*P* < 0.001), stage III (*P* = 0.001), stage IV (*P* < 0.001), and tumor-infiltrating CD8+ T cells (*P* < 0.001) which were completely negative in the kidney cancer development (Figure 7A). Other tumor-infiltrating cells, such as CD4+ T cells, B cells and dendritic cells (all *P* > 0.1) had no significant effects on kidney cancer (Figure 7A) and other cancer types (Tables S3–S6). Additionally, RADIL gene expression increased significantly in a murine immune-tolerance tumor cell line when compared with the murine immune-susceptible tumor cell line (Figure 7B) (17), suggesting RADIL may play a key role in tumor immune resistance.

## Discussion

Early detection and treatment are critical for *de novo* malignancies in renal recipients. In the current study, RADIL and APTX were developed as the two-gene prognostic model for diagnosis for all 42 subjects. Our data revealed that RADIL had a similar AUC value with that of the 2-gene signature model. Through the comparison between RADIL and APTX, we found that RADIL was a stronger predictor for diagnosis. We also found that while renal, liver, and stomach cancer patients had high expression of RADIL, pancreatic cancer patients had lower expression of RADIL. Immunohistochemistry results showed a similar outcome to that of survival analysis, suggesting RADIL's different roles in various tumor types. The mechanism is unclear and requires further study. We also found that RADIL had a similar trend to the entire cohort in renal, liver, stomach, and pancreatic cancer in the early stages (I and II). RADIL could therefore be helpful in predicting the OS of renal, liver and stomach patients in stage I and II. Furthermore, RADIL had a significant effect on accelerating the development of kidney cancer and a negative relationship with tumor-infiltrating CD8+ T cells. In addition, RADIL gene expression increased significantly in murine immune-tolerance tumor cell lines.

Previous studies demonstrate that RADIL can bind to RAP1A in a GTP-dependent manner and plays a key role in cell adhesion or migration (18, 19). Moreover, RAP1A-RADIL inside-out signaling which is essential for breast cancer cell migration and invasion, can be inhibited by KLF14 through binding to C-terminal PDZ domain of RADIL and restricting RADIL from interacting with activated RAP1A spatially (9). Research done by groups at National Cancer Institut has shown that RADIL can regulate neutrophil adhesion and chemotaxis by RADIL-mediated integrin and focal adhesion kinase (FAK) activation (20), which has been reported as a potentially important new target for cancer therapy (21). In the current study, we found that RADIL had a significant difference between non-malign and malign groups. These results support the hypothesis that RADIL has the ability to control the key genes in the process of tumor development. Compared with the previous study, RADIL had a higher ROC area than IL27 (0.91 VS 0.88) though IL27 is an adequate predictor of malignancy after kidney transplantation (data not shown) (5). As a DNA repair-related protein, APTX can promote the repair of DNA single strand breaks caused by various DNA damaging agents (10). A previous report showed that targeted inactivation of APTX significantly sensitized cells to camptothecin (an anti-cancer drug) treatment in colorectal cancer cell lines. Low APTX tumor levels were associated with good response to irinotecan (another anti-cancer drug) in colorectal cancer patients (11). While in the current study, the AUC of APTX proved not optimal in the test group, these results suggest APTX may not be appropriate for predicting post-Tx kidney cancer.

Recently, the Eric group followed 16,820 kidney transplant recipients, among which 923 died from cancers - a 2.5-fold higher mortality rate than the general population. Kidney cancer in post-Tx recipients has been the second highest cause of death and the standardized mortality ratios were 7.8% (22). In the current study, though we found RADIL had a better AUC in all stages of kidney, liver, stomach, and pancreatic cancer, Cox regression analysis found that RADIL is a significant risk factor for the development of kidney cancer. The analysis also found tumor-infiltrating CD8+ T cells may suppress the development of kidney cancer. CD8+ cytotoxic T cells are a major adaptive effector cell subset which can destroy both allografts and tumor cells (7, 23). The administration of post-Tx immunosuppressants keeps the recipients immunosuppressed for an extended period of time, resulting in decreased CD8+ cytotoxic T cells and low immune surveillance function, increasing susceptibility to viral infection and tumor incidence. Kroemer group has proven the application of Cyclosporine A can promote melanoma growth by downregulating the Th1 hallmark T-box transcription factor T-bet in OTI cytotoxic T cells in a skin graft model (7). However, there was no significant difference in tumor-infiltrating memory T cells, which may be caused by the differentiation of CD8+ OTI.T-bet^−^/^−^ cells into Tc17 T cells. In the current study, we found that CD8+ T cells had a negative function in kidney cancer development, which was consistent with the previous study (23). In addition, previous studies have reported the migration of tumor-infiltrating cluster CD8+ T cells was inhibited significantly in immune-resistant human renal cell carcinoma (RCC). RADIL gene expression was significantly upregulated when immune resistance occurred, which could prove that RADIL could accelerate the RCC development when tumor cells escape from the CD8+ immune surveillance (17).

Though we confirmed the significant prognosis role of RADIL in the peripheral blood in kidney cancer occurrence and development after kidney transplantation, the research is limited because of the small number of kidney transplant recipients, the retrospective nature of the study, and the unclear underlying mechanisms. Ultimately, the prognostic potential of RADIL needs further study in more post-Tx recipients.

## Conclusion

We have identified RADIL as an accurate diagnosis and prognosis biomarker for kidney cancer occurrence and development at peripheral blood level in the current cohort. We are currently working on the RADIL-relevant mechanisms which may be closely related to tumor-infiltrating CD8+ T cells. This research may provide a new immunotherapy target for predicting post-Tx kidney cancer.

## Data Availability

The datasets generated for this study are available on request to the corresponding author.

## Author Contributions

QF, FY, and ML: study design. FY and ML: sample and data acquisition. LW, HY, and KC: statistical analysis. QF, HY, KC, and JM: drafting of the manuscript. NF, KD, and NS: revising of the manuscript. LW and SD: obtained funding. All authors reviewed the manuscript.

### Conflict of Interest Statement

The authors declare that the research was conducted in the absence of any commercial or financial relationships that could be construed as a potential conflict of interest.

## Abbreviations

APTX: Aprataxin
AUC: Area under the curve
AOD: Average optical density
CAMR-Tx: Chronic antibody-mediated rejection after transplantation
CTRL-Tx: Control transplant
Tc: Cytotoxic T
DEGs: Differentially expressed analysis
ESRD: End-stage renal disease
FAK: Focal adhesion kinase
OS: Overall survival
PBMC: Peripheral blood mononuclear cell
post-Tx: Post-transplant
PCA: Principal component analysis
RADIL: Rap GTPase Interactor
RCC: Renal cell carcinoma
ROC: Receiver operating characteristic
TCGA: The Cancer Genome Atlas
TNM: Tumor node metastasis.
