# Supplementary material for: Rap GTPase Interactor: A Potential Marker for Cancer Prognosis Following Kidney Transplantation
Source: Front Oncol. 2019 Aug 7;9:737. doi: 10.3389/fonc.2019.00737 (PMC6692533; doi:10.3389/fonc.2019.00737)
Supplement: Supplementary file 1 [file Data_Sheet_1.docx]

| Demographic and clinical characteristics | Microarray group (Training group) | | | Microarray group (Test group) | | |
| --- | --- | --- | --- | --- | --- | --- |
|  | CTRL Tx | Post-Tx Malignancies | Healthy subjects | CAMR | Non Tx malignancies | *P* value |
| Patients, n | 8 | 8 | 8 | 10 | 8 | NS |
| Gender (M/F), n/n | 5/3 | 6/2 | 5/3 | 4/6 | 6/2 | NS |
| Age (years), mean ± SD | 52 ± 8.9 | 59.8 ± 8.0 | 53.5 ± 4.6 | 46.8 ± 11.58 | 65 ± 4.0 | NS |
| Tx vintage (months), mean± SD | 80.4 ± 8.4 | 109.2 ± 48.0 |  | Not available |  | NS |
| Cyclosporine, n | 1/8 | 5/8 |  | 2/10 |  | NS |
| Tacrolimus, n | 7/8 | 3/8 |  | 8/10 |  | NS |
| mTOR inhibitor, n | 0/8 | 0/8 |  | 0/10 |  | NS |

**Table S1 Demographic data characteristics of the patients from GEO**

**Table S2 Demographic data characteristics of the patients from protein atlas**

| Demographic and clinical characteristics | Renal cancer-negative | Renal cancer- positive | *P* value | Liver cancer-negative | Liver cancer-positive | *P* value | Stomach cancer-negative | Stomach cancer-positive | *P* value | Pancreatic cancer-negative | Pancreatic cancer-positive | *P* value |
| --- | --- | --- | --- | --- | --- | --- | --- | --- | --- | --- | --- | --- |
| Patient number, n | 4 | 6 | NS | 6 | 5 | NS | 4 | 7 | NS | 3 | 7 | NS |
| Gender (M/F), n | 3/1 | 2/4 | NS | 2/4 | 3/2 | NS | 2/2 | 4/3 | NS | 2/1 | 4/3 | NS |
| Age (years) | 69.25 ± 3.66 | 67.00 ± 2.956 | NS | 65.67 ± 2.951 | 64.60 ± 6.99 | NS | 70.00 ± 1.581 | 76.29 ± 5.584 | NS | 66.00 ± 1.732 | 67.29 ± 1.782 | NS |

|  |
| --- |

Table S3

Cox Proportional Hazard Model of kidney cancer

415 patients with 123 deceased

| Demographic and clinical characteristics | coef | HR | 95%CI_l | 95%CI_u | *P* value | sig |
| --- | --- | --- | --- | --- | --- | --- |
| Age | 0.033 | 1.033 | 1.016 | 1.051 | < 0.001 | *** |
| Gender (Male) | -0.076 | 0.927 | 0.623 | 1.378 | 0.707 |  |
| Race (Black) | 0.033 | 1.034 | 0.128 | 8.353 | 0.975 |  |
| Race (White) | 0.024 | 1.024 | 0.139 | 7.572 | 0.981 |  |
| Stage2 | 0.52 | 1.682 | 0.838 | 3.373 | 0.143 |  |
| Stage3 | 0.818 | 2.265 | 1.398 | 3.668 | 0.001 | ** |
| Stage4 | 1.867 | 6.469 | 4.037 | 10.367 | < 0.001 | *** |
| Purity | 0.04 | 1.04 | 0.441 | 2.455 | 0.928 |  |
| B cell | -0.196 | 0.822 | 0.014 | 46.902 | 0.924 |  |
| CD8+T cell | -2.505 | 0.082 | 0.011 | 0.593 | 0.013 | * |
| CD4+T cell | -2.317 | 0.099 | 0.002 | 4.323 | 0.23 |  |
| Macrophage | -0.689 | 0.502 | 0.031 | 8.251 | 0.63 |  |
| Neutrophil | 2.47 | 11.822 | 0.044 | 3191.016 | 0.387 |  |
| Dendritic | 1.281 | 3.599 | 0.369 | 35.079 | 0.27 |  |
| RADIL | 0.308 | 1.361 | 1.106 | 1.675 | 0.004 | ** |

| Rsquare= 0.202 (max possible= 9.59e-01) |
| --- |
| Likelihood ratio test p= 2.05e-13 |
| Wald test p= 4.69e-13 |
| Score (logrank) test p= 4.44e-16 |

Table S4

| Demographic and clinical characteristics | coef | HR | 95%CI_l | 95%CI_u | *P* value | sig |
| --- | --- | --- | --- | --- | --- | --- |
| Age | 0.014 | 1.014 | 0.997 | 1.032 | 0.113 |  |
| Gender (Male) | -0.02 | 0.98 | 0.601 | 1.597 | 0.935 |  |
| Race (Black) | 1.137 | 3.118 | 1.139 | 8.532 | 0.027 | * |
| Race (White) | -0.091 | 0.913 | 0.552 | 1.511 | 0.724 |  |
| Stage2 | 0.213 | 1.238 | 0.729 | 2.102 | 0.43 |  |
| Stage3 | 0.743 | 2.102 | 1.278 | 3.457 | 0.003 | ** |
| Stage4 | 1.442 | 4.229 | 1.222 | 14.64 | 0.023 | * |
| Purity | 1.082 | 2.951 | 0.975 | 8.931 | 0.055 | · |
| B cell | -6.16 | 0.002 | 0 | 3.757 | 0.107 |  |
| CD8+T cell | -5.767 | 0.003 | 0 | 0.515 | 0.027 | * |
| CD4+T cell | -8.74 | 0 | 0 | 0.395 | 0.028 | * |
| Macrophage | 8.665 | 5798.876 | 20.375 | 1650429.366 | 0.003 | ** |
| Neutrophil | -1.122 | 0.326 | 0 | 46700.337 | 0.853 |  |
| Dendritic | 5.117 | 166.81 | 3.437 | 8095.522 | 0.01 | * |
| RADIL | 0.081 | 1.084 | 0.906 | 1.298 | 0.377 |  |

Cox Proportional Hazard Model of Liver cancer

305 patients with 101 deceased

| Rsquare= 0.166 (max possible= 9.91e-01 ) |
| --- |
| Likelihood ratio test p= 1.23e-02 |
| Wald test p= 4.96e-02 |
| Score (logrank) test p= 4.46e-02 |

Table S5

Cox Proportional Hazard Model of Stomach cancer

| Demographic and clinical characteristics | coef | HR | 95%CI_l | 95%CI_u | *P* value | sig |
| --- | --- | --- | --- | --- | --- | --- |
| Age | 0.039 | 1.039 | 1.018 | 1.062 | < 0.001 | *** |
| Gender (Male) | 0.258 | 1.295 | 0.834 | 2.01 | 0.249 |  |
| Race (Black) | 0.481 | 1.618 | 0.646 | 4.054 | 0.305 |  |
| Race (White) | 0.18 | 1.197 | 0.716 | 2.002 | 0.493 |  |
| Stage2 | 0.753 | 2.123 | 0.929 | 4.847 | 0.074 | · |
| Stage3 | 1.136 | 3.114 | 1.443 | 6.719 | 0.004 | ** |
| Stage4 | 1.472 | 4.359 | 1.52 | 12.498 | 0.006 | ** |
| Purity | -0.439 | 0.645 | 0.286 | 1.452 | 0.289 |  |
| B cell | 5.187 | 178.919 | 1.398 | 22897.956 | 0.036 | * |
| CD8+T cell | -0.711 | 0.491 | 0.021 | 11.663 | 0.66 |  |
| CD4+T cell | -5.534 | 0.004 | 0 | 1.197 | 0.058 | · |
| Macrophage | 6.428 | 618.851 | 9.896 | 38698.466 | 0.002 | ** |
| Neutrophil | -0.837 | 0.433 | 0 | 1026.276 | 0.833 |  |
| Dendritic | 0.433 | 1.542 | 0.072 | 32.822 | 0.781 |  |
| RADIL | 0.142 | 1.152 | 0.993 | 1.338 | 0.062 | · |

278 patients with 104 deceased

| Rsquare= 0.146 (max possible= 9.76e-01) |
| --- |
| Likelihood ratio test p= 1.14e-04 |
| Wald test p= 1.45e-04 |
| Score (logrank) test p= 7.03e-05 |

Table S6

Cox Proportional Hazard Model of Stomach cancer

165 patients with 89 deceased

| Demographic and clinical characteristic | coef | HR | 95%CI_l | 95%CI_u | *P* value | sig |
| --- | --- | --- | --- | --- | --- | --- |
| Age | 0.028 | 1.028 | 1.005 | 1.05E+00 | 0.015 | * |
| Gender (Male) | -0.259 | 0.772 | 0.495 | 1.20E+00 | 0.253 |  |
| Race (Black) | -0.193 | 0.825 | 0.171 | 3.97E+00 | 0.81 |  |
| Race (White) | 0.407 | 1.503 | 0.567 | 3.99E+00 | 0.413 |  |
| Stage2 | 0.134 | 1.144 | 0.463 | 2.83E+00 | 0.771 |  |
| Stage3 | -0.945 | 0.389 | 0.046 | 3.32E+00 | 0.388 |  |
| Stage4 | -0.39 | 0.677 | 0.131 | 3.49E+00 | 0.641 |  |
| Purity | -1.011 | 0.364 | 0.133 | 9.92E-01 | 0.048 | * |
| B cell | 2.8 | 16.438 | 0.035 | 7.64E+03 | 0.372 |  |
| CD8+T cell | 2.45 | 11.583 | 0.016 | 8.60E+03 | 0.468 |  |
| CD4+T cell | -6.531 | 0.001 | 0 | 2.68E+00 | 0.089 | · |
| Macrophage | -3.832 | 0.022 | 0 | 1.84E+01 | 0.265 |  |
| Neutrophil | 12.169 | 192698.597 | 0.14 | 2.65E+11 | 0.092 | · |
| Dendritic | -2.462 | 0.085 | 0.002 | 2.94E+00 | 0.173 |  |
| RADIL | -0.131 | 0.877 | 0.725 | 1.06E+00 | 0.175 |  |

| Rsquare= 0.166 (max possible= 9.91e-01) |
| --- |
| Likelihood ratio test p= 1.23e-02 |
| Wald test p= 4.96e-02 |
| Score (logrank) test p= 4.46e-02 |


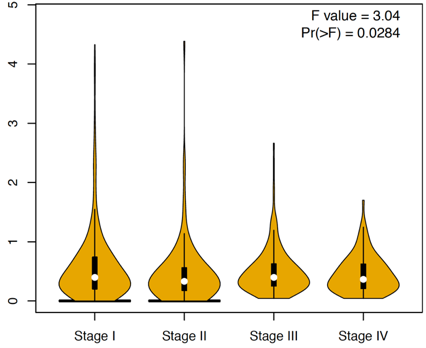


**Figure S1**. RADIL expression was significantly different among all stages in kidney cancers (*P* < 0.05).


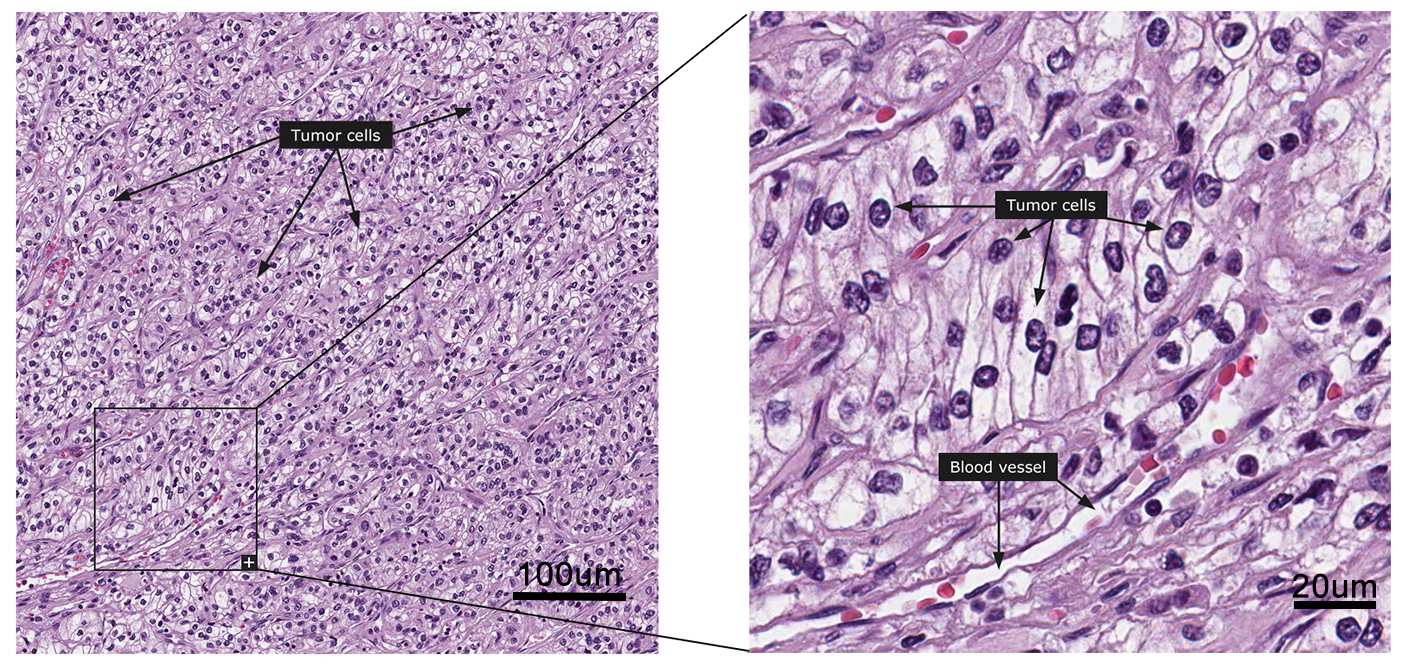


**Figure S2**. The histopathology of kidney cancer.
